# Clinical Implications of the 2025–2030 Dietary Guidelines for Americans for Diabetes and Prediabetes Management

**DOI:** 10.1111/1753-0407.70234

**Published:** 2026-05-13

**Authors:** Yoriko Heianza, Frank B. Hu

**Affiliations:** ^1^ Department of Epidemiology, Celia Scott Weatherhead School of Public Health and Tropical Medicine Tulane University New Orleans Louisiana USA; ^2^ Department of Nutrition Harvard T.H. Chan School of Public Health Boston Massachusetts USA; ^3^ Department of Epidemiology Harvard T.H. Chan School of Public Health Boston Massachusetts USA

## Abstract

- The 2025–2030 DGAs emphasize whole, minimally processed foods while recommending reductions in ultra‐processed foods, refined carbohydrates, added sugars, and sugar‐sweetened beverages, aligning well with diabetes nutrition therapy.
- The DGAs retain a limit of ⟨10% energy from saturated fat, yet promote animal‐derived foods high in saturated fat (e.g., full‐fat dairy, butter), creating a potential inconsistency for cardiovascular risk management.
- The DGAs recommend higher protein intake (1.2–1.6 g/kg/day) with an emphasis on animal protein sources, which may be concerning for kidney health, particularly in individuals with or at risk for chronic kidney disease.
- The DGAs should be applied with individualized nutrition therapy, tailoring dietary patterns to patients’ metabolic status, comorbidities, and preferences.

## 1 Introduction

Lifestyle changes, including dietary modifications, can reduce the risk of developing type 2 diabetes by approximately 40%–50% in high‐risk adults with prediabetes [1]. Individualized medical nutrition therapy (MNT) is an essential component of diabetes management and is associated with significant reductions in HbA1c levels in individuals with type 1 or type 2 diabetes [2]. The 2025–2030 Dietary Guidelines for Americans (DGAs), issued by the U.S. Department of Health and Human Services and the U.S. Department of Agriculture [3], provide updated recommendations emphasizing diets built on whole, nutrient‐dense “real foods.” These include high‐quality protein at every meal from a variety of animal and plant sources, healthy fats, colorful vegetables and fruits, and whole grains. The new DGAs also call for a substantial reduction in highly processed foods rich in refined carbohydrates, added sugars, excess sodium, unhealthy fats, and additives [3].

Although developed primarily for U.S. nutrition programs such as school meals, the DGAs also influence clinical nutrition practice more broadly. In this commentary, we discuss the potential implications of these recommendations for diabetes management and prevention, as well as the challenges and considerations involved in their implementation. We also discuss whether the new DGAs align with the American Diabetes Association (ADA) Standards of Care Nutrition Recommendations [2]. As summarized in Table 1, the core components of the 2025–2030 DGAs align broadly with the ADA MNT principles, particularly regarding whole foods, fiber‐rich carbohydrates, and reduced added sugars. However, certain recommendations, especially those related to animal‐derived saturated fats and elevated protein targets, are not fully aligned with cardiovascular and kidney risk considerations, especially in high‐risk individuals.

**TABLE 1:** Clinical implications of the 2025–2030 Dietary Guidelines for Americans for diabetes and prediabetes management.

2025–2030 DGA recommendations	Alignment with ADA medical nutrition therapy (MNT)	Clinical implications for diabetes	Areas requiring individualization
Emphasize whole, minimally processed foods	Broadly	Improves overall diet quality, glycemic control, and cardiometabolic risk	Food access, affordability, cultural tailoring
Increase vegetables, whole fruits, legumes, and whole grains	Broadly	Enhances fiber intake; lowers glycemic load; improves insulin sensitivity	Individual carbohydrate tolerance; glycemic monitoring
Limit added sugars and refined carbohydrates	Broadly	Reduces postprandial hyperglycemia and triglycerides	Role of non‐nutritive sweeteners in selected patients; behavioral sustainability
Reduce ultra‐processed foods	Broadly	May decrease excess calorie intake and improve metabolic health	Heterogeneity within ultra‐processed food categories
Limit saturated fat (should not exceed 10% of total daily calories)	Broadly	Supports LDL cholesterol reduction and cardiovascular risk management	Replacement nutrient (PUFA vs. refined carbohydrate); lipid profile
Highlight red meat and animal‐derived fats (e.g., butter, beef tallow) within dietary pattern	Limited	May fit within caloric and macronutrient targets but high in saturated fat	Baseline CVD risk, LDL cholesterol levels, substitution with plant‐based proteins and PUFA, and overall dietary pattern
Increase protein at each meal (1.2–1.6 g/kg/day)	Limited	May enhance satiety and support weight management	CKD status; protein source (plant vs. animal); cardiovascular risk
Recommend 3 servings/day of full‐fat dairy (without added sugars)	Limited	Contributes protein and micronutrients but increases saturated fat intake	CVD risk profile, dyslipidemia, lactose intolerance, overall dietary fat pattern
Visual messaging	Limited	The inverted food pyramid visually emphasizes animal‐source foods	Clarify alignment between visual guidance and individualized food choices

Abbreviations: ADA, American Diabetes Association; CKD, chronic kidney disease; CVD, cardiovascular disease; DGA, dietary guidelines for Americans; LDL, low‐density lipoprotein; MNT, medical nutrition therapy; PUFA, polyunsaturated fatty acids.

## 2 Foods to Avoid or Limit: Moving Beyond Highly Processed Foods

The new DGAs explicitly emphasize limiting highly processed foods, added sugars, refined carbohydrates, and sugar‐sweetened beverages (Table 1). They also recommend restricting sodium intake to less than 2300 mg/day and limiting saturated fat to less than 10% of total daily calories. Overall, the DGAs and ADA recommendations are largely concordant regarding foods to limit or avoid. The ADA's Standards of Care [2] similarly recommend limiting intake of foods high in saturated fat to reduce cardiovascular disease risk. However, some differences remain; for example, while non‐nutritive sweeteners are discouraged in the DGAs, they remain an option to replace added sugars in diabetes management by the ADA.

The National Health and Nutrition Examination Survey data show that ultra‐processed food consumption increased from 2001–02 to 2017–18 among adults with prediabetes and diabetes; the proportion consuming ≥ 75% of daily calories from ultra‐processed foods substantially increased from 12.4% to 21.4% in adults with prediabetes and from 8.9% to 20.5% in those with diabetes [4]. Ultra‐processed food consumption is consistently associated with a higher risk of type 2 diabetes and its complications [5, 6]. High intakes of these foods, along with low‐quality carbohydrates and saturated fats, remain a significant concern among U.S. adults [4, 7]. Preferences for foods high in sugar and fat may exhibit addictive‐like properties mediated by complex biological pathways involving gut–brain reward circuits [8]. The simultaneous combination of fat and sugar may enhance dopamine release, inducing a subconscious drive to overconsume energy‐dense, obesogenic foods [9]. Avoiding highly processed foods (often high in added sugars, sodium, and unhealthy fats) may help downregulate these circuits and support healthier eating habits. The new DGAs also recommend limiting foods and beverages containing artificial flavors, petroleum‐based dyes, preservatives, and low‐calorie non‐nutritive sweeteners. The 2023 World Health Organization guidelines advised against the use of non‐sugar sweeteners for weight control or reducing the risk of non‐communicable diseases [10]. In clinical practice, however, replacing sugar‐sweetened beverages with non‐nutritive alternatives may still provide short‐term glycemic benefit for some individuals, underscoring the need for individualized guidance, as recommended by ADA's MNT.

## 3 Implications of a Shift Toward Whole, Nutrient‐Dense “Real Foods” for Diabetes and Prediabetes

Paired with a reduction in highly processed foods, the new DGAs emphasize diets based on whole, nutrient‐dense foods, including protein foods at every meal, vegetables and fruits throughout the day, fiber‐rich whole grains, dairy, and healthy fats. For cooking, the new DGAs specifically recommend olive oil, but also include butter or beef tallow as “healthy fats” options. Within a 2000‐kcal/day diet, recommended intake levels include approximately three servings/day of dairy (especially full‐fat dairy with no added sugars), three servings/day of vegetables, two servings/day of fruits, and two to four servings/day of whole grains (adjusted as needed based on individual caloric requirements).

Nutrient‐dense foods (i.e., foods high in micronutrients and relatively low in calories, such as vegetables, fruits, and legumes [2]) are also emphasized in ADA recommendations. The ADA's MNT supports eating patterns that include non‐starchy vegetables, whole fruits, legumes, lean protein sources, whole grains, nuts, seeds, and low‐fat dairy or non‐dairy alternatives. To incorporate healthy fats from whole‐food sources, the inverted food pyramid highlights full‐fat dairy, meat, poultry, and eggs, in addition to heart‐healthy fat sources (e.g., omega‐3–rich seafood, nuts, seeds, olives, and avocados). In this context, the broader endorsement of animal‐based foods in the new DGAs is less consistent with cardiovascular risk reduction strategies for individuals with diabetes.

Epidemiological studies have demonstrated differential cardiometabolic effects across these food sources. For milk consumption, among men and women with a diagnosed type 2 diabetes from the Nurses' Health Study and the Health Professionals Follow‐Up Study, a pooled relative risk of mortality was 0.88 (95% CI: 0.80–0.96) for low‐fat milk, and 1.20 (0.99–1.44) for full‐fat milk when comparing the highest with the lowest intake [11]. One cup of whole milk contains approximately 4.5 g of saturated fat. In the implementation of the new DGAs, considering saturated fat targets (which should not exceed 10% of total daily calories or about 22 g in a 2000‐cal diet) alongside the recommended intake of full‐fat dairy (e.g., three servings/day) may limit flexibility in selecting other fat sources. For individuals with diabetes at elevated cardiovascular risk, fat quality, and food substitution patterns require careful consideration. The ADA's Standards of Care emphasize evidence‐based eating patterns, such as the Mediterranean and low‐carbohydrate diets that incorporate plant‐based protein sources (e.g., nuts, seeds, and legumes), to prevent diabetes and reduce cardiovascular risk [2].

## 4 Protein Considerations in Diabetes: Does the Source Matter?

The new DGAs recommend high‐quality, nutrient‐dense protein foods at every meal as part of a healthy dietary pattern, with a reference intake of 1.2–1.6 g/kg body weight/day. Acute protein intake at a meal contributes to appetite regulation [12], and adding dairy or plant protein to a carbohydrate‐containing meal elicits reductions in postprandial glucose excursions in individuals without diabetes [13]. A meta‐analysis of randomized controlled trials showed that single meals containing protein suppress hunger and prospective food consumption, while increasing fullness and satiety, accompanied by decreases in ghrelin and increases in cholecystokinin and glucagon‐like peptide‐1 (GLP‐1) levels [12]. However, there is no evidence that long‐term high‐protein intake beyond recommended levels confers additional cardiometabolic benefits. Although short‐term effects on satiety and glycemic control are well documented, long‐term health outcomes likely depend more on overall dietary pattern and protein source than on protein quantity alone.

While the new DGAs recommend a variety of protein foods from both animal sources (e.g., red meat, eggs, poultry, and seafood) and plant‐based sources, animal protein foods such as meat and full‐fat dairy are more prominently emphasized in the inverted food pyramid. A large body of evidence has demonstrated differential cardiometabolic outcomes depending on protein source, with red meat and animal protein intake generally associated with a higher risk of type 2 diabetes in a dose‐dependent manner [14, 15]. Replacing animal protein, particularly red meat, with plant protein sources is associated with a lower risk of type 2 diabetes [14]. Protein sources vary in amino acid composition, heme iron content, and inflammatory potential, all of which may influence the risk of type 2 diabetes, independent of saturated fat content [16]. Gut microbial metabolism of nutrients from animal proteins, particularly red meat, generates potentially atherogenic metabolites such as trimethylamine N‐oxide [17]. Conversely, the cardiometabolic effects of atherogenic microbial metabolites derived from animal‐based nutrient precursors could be partially attenuated by overall diet quality rich in plant foods [18, 19], underscoring the importance of protein intake within a healthy dietary pattern. Another consideration is that the recommended protein intake is relatively high (1.2–1.6 g/kg body weight). Evidence remains inconclusive regarding the optimal amount of protein to improve glycemic control and reduce cardiovascular risk in people with diabetes [2]. The ADA recommends a lower intake (approximately 0.8 g/kg/day) for individuals with non‐dialysis‐dependent stage G3 or higher chronic kidney disease (CKD) [20]. Given that fewer than 15% of adults with CKD are aware of their diagnosis [21], higher protein recommendations may inadvertently increase the risk of adverse kidney outcomes in susceptible individuals.

## 5 Clinical Implications

MNT is a fundamental component of achieving diabetes management goals and improving the quality of life for individuals with diabetes. Given the heterogeneity of diabetes phenotypes, comorbidities, cultural preferences, and adherence, there is no ideal macronutrient distribution for all individuals with diabetes. Instead, macronutrient intake should be individualized through MNT, which offers a comprehensive and tailored approach. For carbohydrate intake, the ADA emphasizes minimally processed, nutrient‐dense, high‐fiber sources (e.g., vegetables, legumes, whole fruits, and whole grains), with a recommended fiber intake of at least 14 g/1000 kcal [2]. Many core principles of the 2025–2030 DGAs align well with MNT, including prioritizing whole, minimally processed foods; increasing intake of vegetables, whole grains, and legumes; and limiting added sugars and refined carbohydrates. However, broader endorsement of animal‐derived saturated fat sources and higher protein targets by the DGAs is not fully aligned with cardiovascular and kidney risk considerations in high‐risk individuals. For individuals using GLP‐1 receptor agonists, a modest increase in protein intake may be appropriate to help preserve muscle mass during weight loss, although overall diet quality remains central to long‐term cardiovascular outcomes.

In this context, clinicians should emphasize dietary patterns supported by outcome‐based evidence, such as Mediterranean‐style and DASH‐like diets that prioritize vegetables, fruits, whole grains, legumes, nuts, fish, and unsaturated plant oils, consistent with the ADA's MNT recommendations. Replacing saturated fats with unsaturated fats remains a key strategy for reducing cardiovascular risk. Protein intake should be individualized based on age, kidney function, metabolic status, and treatment goals, rather than uniformly increased. Dietary guidance should be culturally adaptable, sustainable, and aligned with patients' food preferences and socioeconomic circumstances to support long‐term adherence.

## 6 Conclusion

The new DGAs have important implications for diabetes prevention and management. Their emphasis on whole, minimally processed foods, fiber‐rich carbohydrates, and the reduction of added sugars strongly aligns with established principles of diabetes MNT. At the same time, recommendations on animal‐derived saturated fats and substantially elevated protein intake are not fully consistent with optimal cardiovascular and kidney risk management in individuals with diabetes. The extent to which the DGAs contribute to reductions in type 2 diabetes and related complications remains to be evaluated. Careful integration of population‐level dietary guidance with individualized MNT remains essential in clinical practice.

## Author Contributions

Y.H. and F.B.H. contributed to conception, drafting, and revising the manuscript, and approved the final version of the manuscript.

## Funding

Y.H. and F.B.H. received research funding from the National Institutes of Health. Y.H. also received funding from the American Diabetes Association. F.B.H. also received funding from the Analysis Group.

## Ethics Statement

The authors have nothing to report.

## Conflicts of Interest

The authors declare no conflicts of interest.

## Data Availability

Data sharing not applicable to this article as no datasets were generated or analyzed during the current study.
